# Exploring transmission dynamics of the *Sarcoptes scabiei* mite in humans by combining molecular typing and epidemiological variables, the Netherlands 2016–2023

**DOI:** 10.1186/s13071-024-06488-y

**Published:** 2024-10-07

**Authors:** Martijn Vink, Hester Coppoolse, Anneke Bergmans, Meike Wennekes, Suzan Pas, Jane Pattipeilohy-van Ommen, Marieta Braks, Sylvia Bruisten, Annemie Galimont-Collen, Bas Wintermans, Ewout Fanoy

**Affiliations:** 1 Department of Infectious Diseases, Public Health Service (GGD) of Rotterdam-Rijnmond, Rotterdam, the Netherlands; 2 Department of Infectious Diseases, Public Health Service (GGD) of Amsterdam, Amsterdam, the Netherlands; 3 Medical Microbiology & Immunology Brabant & Zeeland (Microvida), Roosendaal, the Netherlands; 4 Department of Medical Microbiology, Radboud University Medical Centre, Nijmegen, The Netherlands; 5 Department of Infectious Diseases, Public Health Service (GGD) of Utrecht, Zeist, the Netherlands; 6 Centre for Infectious Disease Control, National Institute for Public Health and the Environment (RIVM), Bilthoven, the Netherlands; 7 Department of Dermatology, Bravis Hospital, Roosendaal, the Netherlands

**Keywords:** Scabies, *Sarcoptes scabiei*, Mite, Molecular typing, Cytochrome c, *cox1*

## Abstract

**Background:**

Scabies, an infestation of the mite *Sarcoptes scabiei*, has seen an increase in clinical diagnoses in the Netherlands since 2011. This study aimed to analyse PCR-positive *S. scabiei* skin samples through partial genome sequencing and to link findings to patient epidemiological characteristics.

**Methods:**

Skin samples were collected from individuals in the Netherlands between January 2016 and January 2023. On the PCR-positive *S. scabiei* skin samples, partial mitochondrial cytochrome* c* oxidase subunit 1 gene (*cox*1) sequencing was performed to assess genetic variability. Epidemiological information was collected through interviews. We examined associations between *cox*1 subtypes, epidemiological factors and treatment outcomes.

**Results:**

Sequencing results were obtained from 128 patients, with epidemiological information available for 55 (43%) of these patients. Fifteen distinct *cox1* subtypes were identified. Subtype 01 was most prevalent (45%) and present across all age groups and social settings. The remaining subtypes were less common and not consistently found in all contexts. Five clusters were identified, each with identical *cox1* subtypes. Comparative analysis with GenBank sequences revealed genetic similarities with strains from Australia, the USA and China, suggesting the global distribution and transmission of specific subtypes. A substantial proportion (73%) of patients with scabies required multiple treatments to eradicate the infestation, with no subtype-related differences.

**Conclusions:**

This is the first study linking *S. scabiei* sequencing results to patient epidemiological data. Several subtypes clustered in specific geographic regions and social contexts, underscoring localised transmission patterns. Further research with larger sample sizes is needed to enhance our understanding of the transmission of this mite. This study provides valuable insights that will strengthen scabies control efforts.

**Graphical Abstract:**

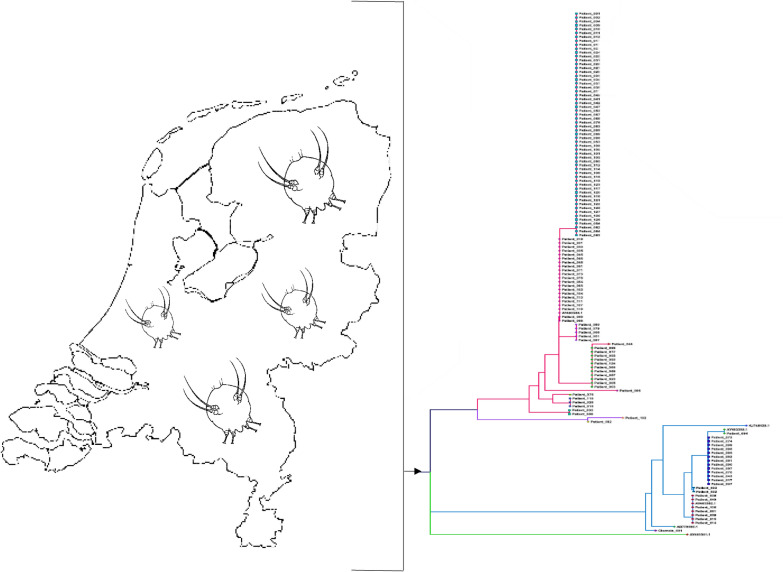

**Supplementary Information:**

The online version contains supplementary material available at 10.1186/s13071-024-06488-y.

## Background

Scabies is a parasitic skin disease caused by the *Sarcoptes scabiei* mite, characterised by skin complaints, such as itching, papules, redness and scaling. Transmission of the mite(s) usually occurs through at least 15 minutes of skin-to-skin contact with a person with scabies, for example during sexual intercourse or by living together, and to a lesser extent through sharing of infested personal items, such as clothes, towels and sheets [1]. Overcrowded living conditions favour the transmission of scabies and can lead to outbreaks, which can be difficult to control. Microscopic examination of skin samples is widely used for the diagnosis of scabies but false negative results are common [2]. More recently, molecular diagnosis using real-time PCR has become available, which has the advantages of a high sensitivity and standardised, relatively easy implementation [3–5].

Individual scabies cases in the general Dutch population are not notifiable except when diagnosed in persons who work in, live in or attend vulnerable settings, such as care institutions or kindergartens [6]. National data sources, including scabies diagnoses reported by Dutch general practitioners (GPs), recorded prescriptions and over-the-counter sales of scabicides, can be used to describe the scabies epidemiology in the Netherlands [7]. Based on a representative sample of Dutch GP practices, the number of scabies diagnoses in the Netherlands increased fourfold between 2011 and 2020, from 0.6 to 2.6 per 1000 persons [7]. The highest incidence was found in the age group between 15 and 29 years [4]. In the same period, the number of prescribed scabicides, including permethrin, benzyl benzoate and ivermectin, increased by more than fivefold [4]. This increase in dispensations was strongly correlated with the increase in scabies diagnoses [7]. The upward trend continued in 2021, with up to 3.3 GP scabies diagnoses per 1000 people [8]. In other European countries, including Germany, France, Norway and Croatia, available data also indicate an upward trend in scabies diagnoses in the last 10 to 20 years [9].

The trend in increasing scabies diagnoses underlines the need to better understand the dynamics of parasitic diseases to better focus control measures. Molecular typing of *S. scabiei* could provide important information for the control of future scabies outbreaks. For example, information on genetic subtypes of *S. scabiei* allows health professionals to determine whether successive outbreaks in the same setting are the result of the introduction of a new subtype or persistence of the same subtype despite the control measures. In the latter case, current control measures may need to be adjusted as the earlier measures may not have been effective. In addition to facilitating direct outbreak control, information on genetic subtypes per social setting would improve understanding of whether transmission cycles in different social settings are interrelated or separate and thereby also require development of adjusted control measures [7].

To date, little is known about the different *S. scabiei* subtypes that circulate worldwide, including in the Netherlands. Only a few studies have used sequencing and typing methods to describe the ecology of different *S. scabiei* subtypes on humans and animals [10]. A recent study used mitochondrial genome sequencing to investigate the genetic diversity within infestations and found limited genetic diversity in single *S. scabiei* infestations [11]. Also, only a limited number of studies have used molecular typing methods in scabies outbreak investigations in humans, in particular for source tracing, detection of extra unknown infestations in transmission chains and evaluation of the effects of interventions [12, 13].

This study is unique in linking partial *S. scabiei* genome sequencing results to epidemiological characteristics of patients. We assessed whether certain *S. scabiei* subtypes are more prevalent in specific epidemiological settings or whether these could be linked to import from abroad. Also, we determined whether certain *S. scabiei* subtypes are associated with infestation persistence, potentially indicating resistance to commonly prescribed scabicides.

## Methods

### Sample collection

In total, 149 *S. scabiei* PCR-positive samples were collected. Between January 2016 and January 2023, Microvida Medical Microbiology Laboratory (Roosendaal, the Netherlands) collected 116 *S. scabiei* PCR-positive skin samples from 116 patients, which were sent in by Dutch dermatologists, medical microbiologists and Public Health Service (PHS) doctors to diagnose scabies. Samples originated individuals from various social contexts, including students, nursing home residents and homeless shelter residents. An additional 30 samples were collected from 12 patients who visited the PHS of Amsterdam (GGD Amsterdam) (2–3 samples per patient collected on the same day as part of a PCR diagnostic validation). Three samples were obtained from one *S. scabiei* PCR-positive piece of skin from a Northern chamois (*Rupicapra rupicapra*) from Italy, provided by the Department of Veterinary Sciences, University of Turin, Italy, as a control. In addition, 17 *S. scabiei* (diagnostic) internal transcribed spacer 2 (ITS2) PCR-negative skin samples were collected to determine the specificity of the PCR for cytochrome* c* oxidase subunit 1 gene (*cox*1) typing.

### Collection of epidemiological information

After obtaining patient consent, individuals with PCR-positive skin samples were contacted for a telephone interview. The interview questionnaire included items on demographic characteristics, the possible source of infestation, the possible transmission route and previous scabies treatments. This questionnaire, translated to English, can be found in Additional file 1: Questionnaire. When patients could not be reached or declined the interview, only the date of sampling, postal code and the year of birth were included in the database.

### *Scarcoptes scabiei* DNA extraction and detection

Skin samples sent to the Microvida Medical Microbiology Laboratory were incubated for a duration ranging between 1 and 12 h at 55 °C in 600 µl of MagNA Pure LC Lysis Binding Buffer (Roche, Mannheim, Germany) with 60 µl of proteinase K (20 mg/ml). DNA was isolated using MagNA Pure 96 DNA and Viral NA Large Volume Kit (Roche) on a MagNA Pure 96 isolation robot (Roche). The DNA was eluted in 100 µl of elution buffer and stored at 4 °C until use. Skin samples and eluted swabs of DNA obtained at the GGD Amsterdam were extracted using the isopropanol extraction method as previously reported by van Houdt et al. [14]. DNA was eluted in 50 µl of buffer and stored at 4˚C until use. *S. scabiei* DNA was detected using an internally controlled real-time PCR targeting the ITS2 [15].

### Partial* cox*1 gene sequence analysis

Based on previous research, three target regions were selected for sequence analysis: (i) the mitochondrial cox1 gene (*cox*1); (ii) the ITS2 region; and (iii) the 12S ribosomal RNA (12S rRNA) gene [16–19]. In silico analysis of the DNA sequences of these three target genes within the GenBank nucleotide database revealed the *cox*1 gene to be the most promising target for molecular typing due to its highest variability across *S. scabiei* populations found on humans from different geographic locations [16]. The *cox*1 PCR primers and protocol used in the study were adapted from Andriantsoanirina et al. [16] to produce solely *S. scabiei*-specific *cox*1 sequences. This modification was deemed necessary since these original primers occasionally generated *cox*1 PCR products of human origin when used on nucleic acid extracts of *S. scabiei* PCR-negative or weakly-positive human skin samples.

DNA extracts (5 μl aliquots) from the diagnostic *S. scabiei* real-time PCR-positive skin samples were used for the PCR tests in 25-µl reaction volumes containing 12.5 µl of Phusion Green Hot Start II High-Fidelity PCR Master Mix (2× concentrated; Thermo Fisher, Bleiswijk, the Netherlands), 300 nM each of the adapted forward primers (cox1Fa-A [5′-TTTTTGGACACCCAGAAGTT-3′], cox1Fa-T [5′-TTTTTGGACACCCTGAAGTT-3′] and cox1Fa-G [5′-TTTTTGGACACCCGGAAGTT-3′], 900 nM of the adapted reverse primer cox1Ra (5′-CAGCACCTATAGATAAWACATAATGA-3′) and 2 µl of MgCl_2_ (25 mM). PCR tests were performed on a Veriti thermal cycler (Applied Biosystems, Thermo Fisher Scientific, Bleiswijk, the Netherlands) for 45 cycles at an annealing temperature 59 °C.

The DNA sequences of the PCR products were determined (both forward and reverse) through Sanger sequencing, using 10-µl sequence reactions of a BrilliantDye v1.1 kit (NimaGen, Nijmegen, the Netherlands). After purification, the sequenced PCR products were separated and analysed using a Genetic Analyzer 3500 (Applied Biosystems, Thermo Fisher Scientific). All generated *cox*1 sequences were submitted to the GenBank database (accession numbers: OR044535-OR044663).

### Data analysis

With the adapted *cox*1 primers and protocol, *cox*1 PCR products of 447 bp were generated. Unambiguous *cox*1 sequences were successfully generated from *S. scabiei* PCR-positive samples with cycle threshold (Ct)-values < 35. Partial *cox*1 sequences generated through Sanger sequencing were analysed using CLC Main Workbench version 22.0 software (Qiagen, Hilden, Germany) to create consensus sequences and generate multiple alignments. Phylogenetic trees (neighbour joining, 1000 bootstraps) of partial *cox*1 sequences of 401 bp were also generated using the CLC Main Workbench v22.0 (Qiagen). Different c*ox*1 subtypes were assigned with a threshold of one nucleotide difference.

Partial *cox*1 sequencing data were linked to epidemiological data through a unique patient identifier. Variation in categorical variables was reported with frequencies and proportions, while variation in quantitative variables was presented with medians and interquartile ranges (IQR). Associations between subtypes, the social context of transmission, the mode of transmission and the number of treatments needed to eradicate the infestation were investigated using Chi-squared tests and Kruskal–Wallis tests in STATA version 15.1 (StataCorp, College Station, TX, USA).

## Results

### Evaluation of the molecular typing method using partial *cox*1 sequencing data

The 447-bp *cox-1* PCR products originated exclusively from *S. scabiei* and not from human DNA as confirmed by Sanger sequence analysis. The 401-bp sequences of *S. scabiei* PCR-positive skin samples were used without the primer sequences for phylogeny analysis. In total, 146 DNA extracts originated from *S. scabiei* PCR-positive skin samples of the 128 patients (127 patients from the Netherlands and one 1 patient from Belgium), and three DNA extracts originated from the Northern chamois (*R. rupicapra*) sample from Italy.

The *cox*1 PCR for *S. scabiei* was also performed on DNA extracts from the 17 skin samples that were negative for the (diagnostic) ITS2 PCR target to determine the specificity of the *cox*1 PCR. This analysis included five dermatophyte PCR-positive samples, of which three samples contained *Trichophyton rubrum*, one sample contained *Trichophyton interdigitale* and one sample contained *Trichophyton violaceum*. In total, 16 of these 17 samples yielded the expected negative results, whereas one sample indicated a weak PCR product of approximately 450 bp in the *cox*1 PCR. Sequence analysis of this last product revealed an unambiguous sequence in only one of the two DNA strands, demonstrating 96% homology to *cox*1 sequences of *Penicillium* and *Aspergillus* species. This result was considered to be a false positive *cox*1 PCR signal.

**Fig. 1:**
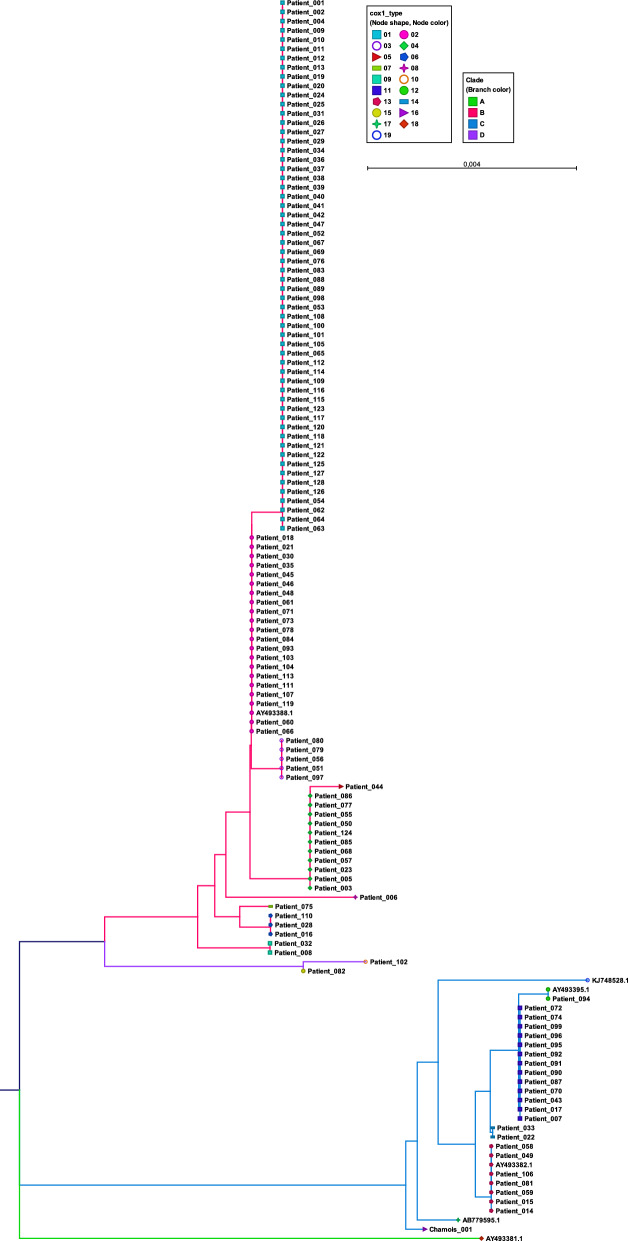
Neighbour joining tree of partial *cox*1 sequences in samples from 128 patients, 1 sample from the Northern chamois (*Rupicapra rupicapra*) and 6 reference sequences from GenBank. Members of each clade (A-D) are shown in different branch colours. Accession numbers: AB779595.1: water buffalo Egypt; AY493381.1: hominis 8 Panama; AY493382.1: hominis 208 Australia; AY493388.1: hominis 10 Australia; AY493395.1: canis 9 USA; KJ748528.1: canis 5 China. *Cox*1, Cytochrome* c* oxidase subunit 1 gene

Fifteen distinctive *cox*1 subtypes were derived from the skin samples of the 128 patients (Fig. 1), with *cox*1 sequence homology varying from 94.3% to 99.8% between the different subtypes. Multiple samples were collected from 12 patients; in 11 of these 12 patients, *cox*1 subtypes from the same patient were identical. This observation also held true for the three samples from the chamois. In one patient from whom three samples were obtained, two samples were *cox*1 subtype 01 and one sample was *cox*1 subtype 02. It should be noted that the *cox*1 subtype 01 differs from subtype 02 by only a single nucleotide.

*Cox1* sequence subtype 01 was found in 45% of patient samples (Table 1). In an attempt to enhance discriminatory power, especially in the large group of samples with *cox*1 subtype 01, we performed sequence analyses on two additional targets: partial ITS2 (approximately 360 bp, 30 specimens) and 12S rRNA gene (451 bp, 32 specimens). However, these sequences showed less polymorphism than the partial *cox*1 gene sequences and no additional discrimination could be made.

**Table 1:** Number of patients and availability of epidemiological information per *Sarcoptes scabiei *cytochrome* c* oxidase subunit 1 (*cox*1) gene subtype

*Cox*1 subtype	Number of patients (*N* = 128)	Percentage of all 128 patients	Number of patients with epidemiological information (*N* = 55)
01	58	45	30
02	21	16	7
03	5	3.9	0
04	11	8.6	5
05	1	0.8	0
06	3	2.3	0
07	1	0.8	1
08	1	0.8	0
09	2	1.6	0
10	1	0.8	1
11	13	10	3
12	1	0.8	1
13	7	5.5	6
14	2	1.6	1
15	1	0.8	0

*Cox1* Cytochrome* c* oxidase subunit 1 gene

### Comparison with* cox*1 sequences from GenBank

Generated *cox*1 sequences were compared to those retrieved from GenBank and to the three genetically distinct clades with monophyletic groups (clade A, B, C) as assigned by Andriantsoanirina et al. [16]. *Cox*1 subtypes 01–09 clustered with a GenBank *cox*1 sequence from a human in Australia (named ‘hominis 10’, accession number AY493388), assigned to clade B (Additional file 2: Figure S1) [16]. *Cox*1 subtype 02 was identical to this Australian sequence. *Cox*1 subtypes 11–14, as well as subtype 16 from the Italian chamois, clustered with a GenBank *cox*1 sequence from another Australian human (‘hominis 208’, accession number AY493382), a dog in the USA (‘canis 9 USA’, accession number AY493395) and a dog in China (‘canis 5 China’, accession number KJ748528). These sequences were all assigned to clade C [16]. Specifically,* cox*1 subtype 12 was identical to the ‘canis 9 USA’ sequence from the USA, and *cox*1 subtype 13 was identical to the ‘hominis 208’ sequence from Australia. C*ox*1 subtypes 10 and 15 were closely related but did not cluster with sequences in clades A, B or C [16].

### Combining* cox*1 sequence subtypes with epidemiological profiles

Among the 128 patients with sequencing results, full epidemiological data were collected for 55 patients (43%) (Table 1); for the remaining 73 patients, only the date of birth and the postal code were available. The median age of the 128 patients was 24 (IQR 19–54) years. Analysis showed that median patient age did not differ significantly between the different *S. scabiei cox*1 subtypes (Kruskal-Wallis test, H = 11.3, df = 14, *P* = 0.66).

Among the 55 patients for whom epidemiological data were available, *cox*1 subtype 01 was found in most of the social contexts, including the student context, healthcare sector and in homeless shelters (Table 2). *Cox*1 subtypes 02, 04 and 13 were dominant in the household/family context, whereas in the school context, *cox*1 subtypes 02 and 10 were dominant. The social context was unknown for four patients (7.2%). The distribution of *cox*1 subtypes was significantly different across social contexts (Chi-square test, χ2 = 98.1, df = 104, *P* = 0.004).

**Table 2:** Association between *Scarcoptes scabiei *cytochrome* c* oxidase subunit 1 (*cox*1) gene subtype and social context where the patient was possibly infested

*Cox*1 subtype	Household or family, *N* (%)	School context^a^, *N* (%)	(Sport) club, *N* (%)	Student context, *N* (%)	Healthcare setting^b^, *N* (%)	Homeless shelter, *N* (%)	Abroad, *N* (%)	Other social context^c^, *N* (%)	Unknown, *N* (%)	Total	*P*-value (Chi-square test)
01			1 (50%)	13 (81%)	4 (80%)	2 (50%)	4 (50%)^d^	5 (63%)	1 (25%)	30	0.004^g^
02	1 (25%)	3 (75%)		2 (13%)				1 (13%)		7	
04	2 (50%)			1 (6%)		1 (25%)			1 (25%)	5	
07							1 (13%)^e^			1	
10		1 (25%)								1	
11			1 (50%)			1 (25%)			1 (25%)	3	
12					1 (20%)					1	
13	1 (25%)						3 (38%)^f^	2 (25%)		6	
14									1 (25%)	1	
Total	4	4	2	16	5	4	8	8	4	55	

^a^Primary/secondary school, school camp or playing with school friends

^b^Includes home-based care

^c^Sexual contact (non-student) (3), child holiday camp (2), holiday home (1), sleeping in someone else’s bed (1), second-hand clothing (1)

^d^Curacao, Denmark, Germany, Peru

^e^Possibly Spain

^f^Turkey (cluster)

^g^Chi-square test, χ^2^ = 98.1, df = 104

Among the 55 patients with epidemiological data, 22 patients (40%) were not aware of the source of transmission, so their transmission routes were unknown. In total, 19 patients (35%) self-reported transmission via skin contact, while nine patients (16%) reported transmission through sexual or intimate contact. Other transmission routes reported included bedding materials (4 patients, 7.3%) and couch pillows (1 patient, 0.6%). There were no significant differences in reported transmission routes for the different *cox*1 subtypes (Chi-square test, χ^2^ = 24.4, df = 52, *P* = 0.83).

### Geographical distribution of* cox*1 subtypes across the Netherlands

**Fig. 2:**
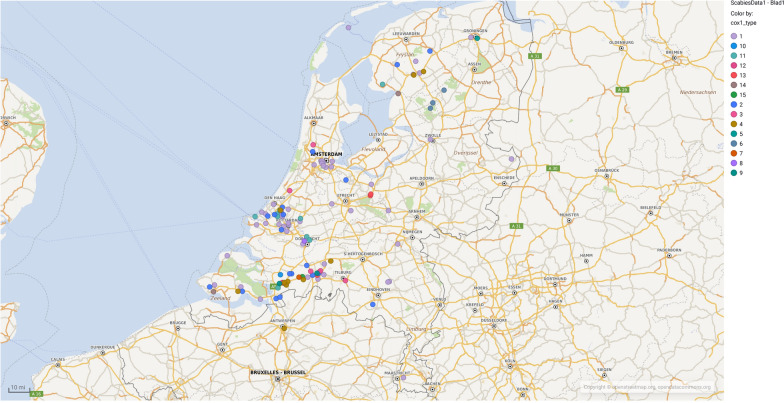
Geographical distribution *Scarcoptes scabiei cox*1 subtypes across the Netherlands. *cox*1, Cytochrome* c* oxidase subunit 1 gene

Using the postal codes of all patients, we linked the *cox*1 subtypes to geographical locations. *Cox*1 subtype 01 and, to a lesser extent, subtype 02 were geographically distributed across the country (Fig. 2). Conversely, *cox*1 subtype 03 was concentrated in the provinces of Noord-Brabant, Zuid-Holland and Noord-Holland; *cox*1 subtypes 04 and 11 were most prevalent in Noord-Brabant, Zuid-Holland and Friesland; and *cox*1 subtype 13 was concentrated in Utrecht and Noord-Brabant. All three patients with *cox*1 subtype 06 were living within a 10- to 20-km radius in the provinces of Overijssel and Drenthe. *Cox*1 subtypes 05, 07, 08, 09, 10, 12, 14 and 15 were limited to one or two locations within the country.

### Association between* cox*1 subtypes and import cases

Eight patients indicated that their *S. scabiei* infestation likely occurred abroad or originated from individuals who had travelled abroad (Table 2). Among these patients, four were infested with *S. scabiei cox*1 subtype 01 while they or their index cases had travelled to Denmark, Germany, Peru or Curaçao. One patient with *cox*1 subtype 04 assumed to have contracted *S. scabiei* during a trip to Spain. Three other patients, all residing in the same household, were infested with *S. scabiei cox*1 subtype 13: one had acquired *S. scabiei* in Turkey and subsequently transmitted it to the two other household members upon return.

### Outbreak clusters

Closer examination of postal codes and *S. scabiei* sources indicated five outbreak clusters in the dataset. Clusters were characterised by cases living in the same household, family, health facility or place of residence with a common *S. scabiei* source. Cases within each of these clusters exhibited identical *S. scabiei cox*1 subtypes, except in one instance. Mites in samples from two young children residing in the same homeless shelter showed distinct *cox*1 subtypes, namely *S. scabiei cox*1 subtype 01 (clade B) and *S. scabiei cox*1 subtype 11 (clade C).

### Association between* cox*1 subtypes and number of treatments

Of the 55 interviewed patients with scabies, 40 patients (73%) required multiple treatments to eliminate the infestation (Table 3). Among those requiring retreatment, 31 patients (78%) received additional ivermectin tablets following their previous permethrin or benzylbenzoate cream treatment. In several cases, initial misdiagnosis by GPs or dermatologists delayed the correct diagnosis and subsequent treatment. We did not find a statistically significant association between the *cox*1 subtype and the number of treatments needed (Chi-square test, χ^2^ = 9.5, df = 13, *P* = 0.30).

**Table 3:** Association between *Scarcoptes scabiei *cytochrome* c* oxidase subunit 1 (*cox*1) gene subtype and number of treatments needed to eliminate the *S. scabiei* infestation

*Cox*1 subtype	Single treatment sufficient, *N* (%)	Multiple treatments necessary, *N* (%)	Total number of patients with epidemiological information	*P*-value (Chi-square test)
01	9 (30%)	21 (70%)	30	0.30^a^
02	1 (14%)	6 (86%)	7	
04	2 (40%)	3 (60%)	5	
07	0	1 (100.0%)	1	
10	1 (100%)	0	1	
11	2 (67%)	1 (33%)	3	
12	0	1 (100%)	1	
13	0	6 (100%)	6	
14	0	1 (100%)	1	
Total	15 (27%)	40 (73%)	55	

^a^ Chi-square test, χ^2^ = 9.5, df = 13

## Discussion

To the best of our knowledge, our study is one of the first studies to link *S. scabiei* genetic typing data with extensive epidemiological patient data on potential sources and transmission routes in humans. Partial sequence analysis on the *S. scabiei* mite from 128 patients with scabies with diverse epidemiological backgrounds in the Netherlands revealed 15 different *S. scabiei cox*1 subtypes. *Cox*1 subtype 01 predominated in patient samples, followed by *cox*1 subtype 02. Regional clustering of most *cox*1 subtypes was observed, and the distribution of *cox*1 subtypes was significantly different across social contexts. Thereby, our epidemiological data in combination with comparative analysis with GenBank sequences suggest a global distribution and transmission of specific *S. scabiei cox*1 subtypes. These findings indicate both international and localised transmission patterns.

The *S. scabiei cox*1 subtypes found in this study, apart from subtypes 10 and 15, are classified within clades B or C [16]. Clade B mites in this study were, based on the patients’ epidemiological information, linked to Europe (the Netherlands, Belgium, Denmark and Germany) and South America (Curacao and Peru), whereas clade C mites were linked to Europe only (the Netherlands and Turkey). Limited additional studies were found to better interpret the global distribution of the scabies mite clades. In a previously published study by Andriantsoanirina et al. [16], clade B mites were found in Europe (France) and Australia and clade C mites were distributed across five continents. These authors also found that clade B exclusively contained mites collected from humans, whereas clade C included mites collected from both humans and animals [16]. Consistent with these findings, our *S. scabiei cox*1 subtype 16 obtained from the Italian chamois clustered with clade C. Moreover, our findings indicated that the DNA sequence of *cox*1 subtype 12, also belonging to clade C, was identical to the GenBank *cox*1 sequence from *S. scabiei* collected from a dog in the USA. This observation regarding the *cox*1 gene is an exception to the hypothesis that humans and animals are infected with distinct *S. scabiei* subtypes [16, 19]. Although it remains unclear whether *S. scabiei* found on the dog had a human index case and was only a temporary carrier, the case does illustrate the potential for shared infestations by *S. scabiei cox*1 subtypes. Additional research is needed to understand the role of pets in the transmission chain. It should further be noted that *cox*1 subtypes 10 and 15 differed substantially from those in clades B and C, prompting the proposal of a new clade with monophyletic groups: clade D.

Our findings underpin the reliability and consistency of results obtained through molecular typing of *S. scabiei* mites. For example, five epidemiological clusters in our dataset showed identical *S. scabiei cox*1 subtypes in connected patients. In contrast, samples from two young children from separate families who resided in the same homeless shelter showed distinct *cox*1 subtypes; it is well possible that these cases contracted their *S. scabiei* from different sources and may not have had direct contact in the homeless shelter. Likewise, *S. scabiei cox*1 subtypes in multiple samples from the same patient were identical for almost all patients. Only one patient donated two samples with *cox*1 subtype 01 and one sample with subtype 02, which differ in only one nucleotide. Samples were retested and the same result was obtained, making laboratory mistakes highly unlikely. The *cox*1 gene enables discrimination between mite clades, but this mutation may have arisen spontaneously in mites found on humans. Alternatively, two or more *S. scabiei* subtypes may have been present in the samples of this person, each with a different *cox*1 subtype.

Several challenges were faced during the implementation of this study. One limitation is the abovementioned small and not very polymorphic region of the *S. scabiei cox*1 gene being used for typing. Several *cox*1 subtypes differ by few or even only one nucleotide(s), so more samples need to be analysed to determine an epidemiological useful typing threshold. In addition, more polymorphic regions in the *S. scabiei* genome need to be identified. Unfortunately, few full *S. scabiei* genomes have been published until now, partly because of the lack of sufficient DNA that is not contaminated with human or animal DNA. Secondly, due to separate legal authorities, we encountered legal challenges when linking molecular data from the microbiology laboratory and epidemiological data from the PHS. This issue was resolved through an ethical waiver. It was also difficult to obtain patient contact details via the referring specialists, and several patients were unwilling to participate due to the time elapsed since the elimination of their infestation. In future studies, patient cooperation and contact information should be obtained during skin sample collection or upon a positive PCR test result, improving data collection efficiency and data quality. Lastly, our sample size with available epidemiological data was relatively small, and additional research with a larger, international sample size is needed to validate our findings and better understand *S. scabiei* transmission dynamics in the Netherlands and abroad.

This study underscores the public health potential of combining *S. scabiei* typing data and epidemiological patient data to better understand transmission patterns and circulation within social contexts. We reported an adapted typing method for *S. scabiei* DNA directly from skin samples that worked in practice, although further efforts are needed to improve its discriminatory power. Insights into transmission chains are informative for focussed scabies control measures needed to respond to the increasing scabies diagnoses observed in the Netherlands and other European countries in the last decade.

## Supplementary Information

**Additional file 1: Questionnaire.** Questionnaire in Dutch was translated to English and used to collect epidemiological data from scabies patients.

**Additional file 2: Figure S1.** Neighbour Joining tree (*n* = 1000 bootstraps) of representatives of the 15 *cox*1 sequence subtypes found in skin samples from 128 patients, 1 chamois and 6 reference sequences from GenBank. Members of each clade (A–D) are shown in different branch colours. Accession numbers: AB779595.1 = water buffalo Egypt; AY493381.1 = hominis 8 Panama; AY493382.1 = hominis 208 Australia; AY493388.1 = hominis 10 Australia; AY493395.1 = canis 9 USA; KJ748528.1 = canis 5 China). Numbers in grey are bootstrap values (%).

## Data Availability

All generated cox1 sequences are available in GenBank repository with the accession numbers OR044535-OR044663.

## Abbreviations

Cox1: Cytochrome* c* oxidase subunit 1
Ct: Cycle threshold
GPs: General practitioners
IQR: Interquartile ranges
PHS: Public Health Service
