# Supplementary material for: Exploring transmission dynamics of the Sarcoptes scabiei mite in humans by combining molecular typing and epidemiological variables, the Netherlands 2016–2023
Source: Parasit Vectors. 2024 Oct 7;17:419. doi: 10.1186/s13071-024-06488-y (PMC11459799; doi:10.1186/s13071-024-06488-y)
Supplement: Supplementary file 1 — Additional file 1: Questionnaire. Questionnaire in Dutch was translated to English and used to collect epidemiological data from scabies patients. [file 13071_2024_6488_MOESM1_ESM.pdf]

Questionnaire epidemiological characteristics of patients with scabies

Epidemiological information

1. Gender
   (0=man, 1=woman, 2=other/unknown)

2. Year of birth

3. Age at diagnosis
   (age in years)

4. Four-digit zip code

5. City of residence

6. (Suspected) direct source
   0=family contact
   1=work
   2=childcare center
   3=student home/association
   4=resident in or employee at care facility or nursing home
   5=resident in or employee at asylum center or refugee center
   6=resident in or employee at homeless shelter
   7=foreign, namely ....
   8=other, namely ....
   9=unknown

7. Certainty of this direct source:
   0=very likely
   1=probably
   2=somewhat likely
   3=source completely unknown

8. (Suspected) initial source
   0=family contact
   1=work

2=childcare center
3=student home/association
4=resident in or employee at care facility or nursing home
5=resident in or employee at asylum center or refugee center
6=resident in or employee at homeless shelter
7=foreign, namely ....
8=other, namely ....
9=unknown

9. Certainty of this original source:
   0=very likely
   1=probably
   2=somewhat likely
   3=source completely unknown

10. Suspected route of infection
    0=via intensive skin contact (sex, hugging, etc.)
    1=via bedding (e.g., lodging)
    2=via shared clothing
    3=other, namely..
    4=unknown

Possible treatment resistance

11. Previous scabies treatment in the past 12 months
    (0=No, 1=Yes)

12. If previous question was answered yes, previous treatment used:
    1=Ivermectin,
    2=Permethrin,
    3=combination of Ivermectin and Permethrin
    4=other treatment

13. Additional details about infection or treatment that may be relevant:
