# Supplementary material for: Exploring transmission dynamics of the Sarcoptes scabiei mite in humans by combining molecular typing and epidemiological variables, the Netherlands 2016–2023
Source: Parasit Vectors. 2024 Oct 7;17:419. doi: 10.1186/s13071-024-06488-y (PMC11459799; doi:10.1186/s13071-024-06488-y)
Supplement: Supplementary file 2 — Additional file 2: Figure S1. Neighbour Joining tree (n = 1000 bootstraps) of representatives of the 15 cox1 sequence subtypes found in skin samples from 128 patients, 1 chamois and 6 reference sequences from GenBank. Members of each clade (A–D) are shown in different branch colours. Accession numbers: AB779595.1 = water buffalo Egypt; AY493381.1 = hominis 8 Panama; AY493382.1 = hominis 208 Australia; AY493388.1 = hominis 10 Australia; AY493395.1 = canis 9 USA; KJ748528.1 = canis 5 China). Numbers in grey are bootstrap values (%). [file 13071_2024_6488_MOESM2_ESM.pdf]

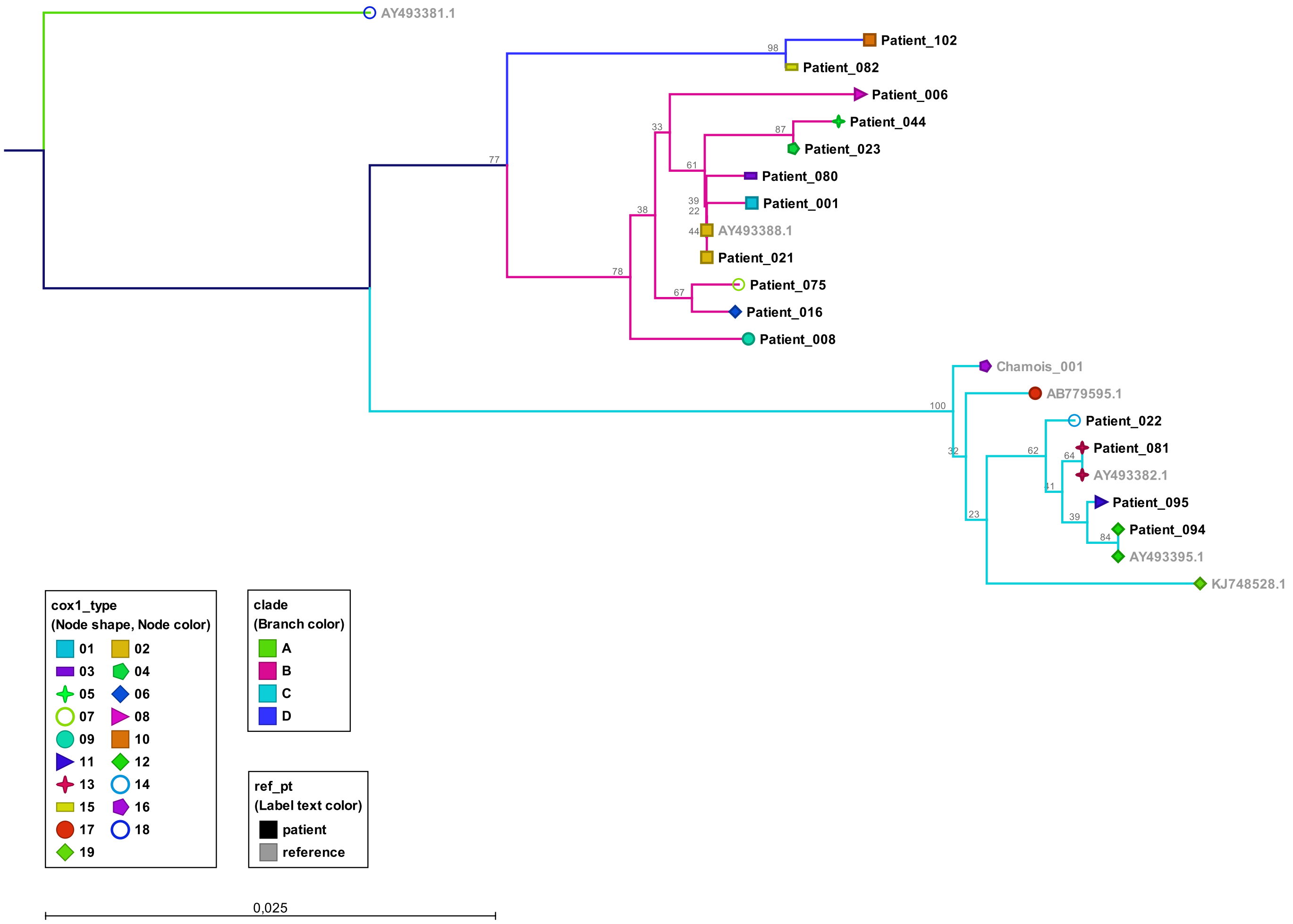
AY493381.1
Patient_102
Patient_082
Patient_006
Patient_044
Patient_023
Patient_080
Patient_001
AY493388.1
Patient_021
Patient_075
Patient_016
Patient_008
Chamois_001
AB779595.1
Patient_022
Patient_081
AY493382.1
Patient_095
Patient_094
AY493395.1
KJ748528.1
98
77
33
87
61
39
22
44
38
78
67
100
62
64
32
41
39
84
23
cox1_type
(Node shape, Node color)
01 02
03 04
05 06
07 08
09 10
11 12
13 14
15 16
17 18
19
clade
(Branch color)
A
B
C
D
ref_pt
(Label text color)
patient
reference
0,025
